# Fluorescence signal of proteins in birch pollen distorted within its native matrix: Identification of the fluorescence suppressor quercetin-3-O-sophoroside

**DOI:** 10.1007/s00216-022-04109-0

**Published:** 2022-05-12

**Authors:** Teresa M. Seifried, Paul Bieber, Victor U. Weiss, Ernst Pittenauer, Günter Allmaier, Martina Marchetti-Deschmann, Hinrich Grothe

**Affiliations:** 1 Institute of Materials Chemistry, TU Wien, 1060 Vienna, Austria; 2 Institute of Chemical Technologies and Analytics, TU Wien, 1060 Vienna, Austria

**Keywords:** Birch pollen, Fluorescence, Inner filter effect, Mass spectrometry, Capillary electrophoresis, UV/Vis

## Abstract

The properties of biogenic aerosol strongly depend on the particle’s proteinaceous compounds. Proteins from primary biological aerosol particles (PBAPs) can cause allergic reactions in the human respiratory system or act as ice and condensation nuclei in clouds. Consequently, these particles have high impact on human health and climate. The detection of biogenic aerosol is commonly performed with fluorescence-based techniques. However, many PBAPs (i.e., pollen of birch, mugwort, or ragweed) show weak or rather low fluorescence signals in the particular protein region (*λ*_ex_ ~ 255–280 nm, *λ*_em_ ~ 280–350 nm). We hypothesize that the fluorescence signal of proteins present in birch pollen is being distorted within its native matrix. In this study, we conducted in vitro quenching experiments and employed UV/Vis spectroscopy, capillary zone electrophoresis (CZE), liquid chromatography (LC), electrospray ionization mass spectrometry (ESI–MS), and multistage MS (MS^2^ and MS^3^) to target major components in birch pollen washing water (BPWW) possibly quenching the fluorescence activity of proteins and thus explaining the lack of corresponding protein fluorescent signals. We identified quercetin-3-O-sophoroside (Q3OS, MW 626 g mol^−1^) to be the main UV/Vis absorbing component in BPWW. Our results point out that Q3OS suppresses the fluorescence of proteins in our samples predominantly due to inner filter effects. In general, when applying fluorescence spectroscopy to analyze and detect PBAPs in the laboratory or the atmosphere, it is important to critically scrutinize the obtained spectra.

**Graphical abstract:**

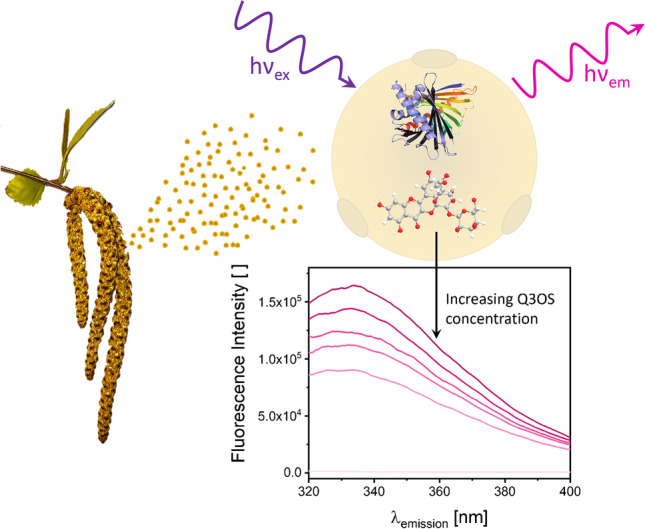

**Supplementary Information:**

The online version contains supplementary material available at 10.1007/s00216-022-04109-0.

## Introduction

Primary biological aerosol particles (PBAPs) are a subcategory of particles released from ecosystems into the atmosphere [1]. One major contributor to PBAPs during springtime are pollen grains from angiosperms (flowering plants) [2]. During the pollination season, the concentrations of pollen grains can reach about 10,000 particles per cubic meter. Pollen act as carrier particle for proteins and other macromolecules with the ability to stay in the atmosphere for several days [3].

Airborne pollen and pollen-attached proteins can be transported long distances (up to 1,000 km horizontally [4], 2–3 km vertically [5]). Some angiosperms release pollen grains leading to allergic reactions in the respiratory tract causing asthma or rhinitis [6, 7]. D’Amato et al. [6] reported that a third of the European population is affected by allergies induced by pollen. Among the most allergenic tree pollen grains are pollen from silver birches (*Betula pendula*) [6]. The major allergen of birch pollen is the protein Betv1a [8, 9] with several isoforms, slightly differing in their amino acid sequences [10]. These isoforms are important for the reproduction of the plant since they function as a fingerprint system, to avoid self-pollination [11].

Birches grow in large stands in the Northern boreal forests, where the plants need to deal with long winters and sub-zero temperatures. To survive frost, the plant (also including its pollen) embodies water-soluble ice-nucleating macromolecules (INMs) [12, 13]. These INMs play a crucial role in the freezing protection mechanism (extracellular freezing). INMs trigger ice crystal formation in extracellular spaces allowing the withdrawal of water from the cell. This leads to an increase in the supercooling capacity of the cell, preventing it from damage [14]. Like proteins, these INMs are likewise part of PBAPs. Burkart et al. [15] claimed that INMs from birch pollen are of proteinaceous origin. Besides INMs protecting the plant from frost damage, they can affect glaciation processes in the atmosphere when getting aerosolized [16]. Seifried et al. [17] showed that INMs from birches can be extracted and splashed off the birch surfaces during rain events. With unoccupied aerial vehicles (UAVs), ice nucleation active material was detected directly above the canopy of a birch forest [18, 19]. In the atmosphere, INMs can act as ice nuclei by initiating the phase transition from liquid water droplets to ice crystals allowing the formation of mixed-phase clouds [20].

Birch pollen itself are about 22 μm in diameter, spherically shaped, and have a density of about 800 kg/m^3^ [21]. Thus, they are unlikely to reach high altitudes where mixed-phase clouds appear due to high settling velocities. However, pollen can fragment or release sub-particles, which are not only an order of magnitude smaller in size (in the range of 1 µm [15, 22]) but also orders of magnitude higher in number concentration compared to their mother grains [23]. In addition, these fragments can still act as protein and macromolecule carriers [15]. A transport model from Werchner [24] shows that sub-pollen particles of birches can be transported further compared to their mother pollen grains. Thus, allergens and INMs can be transported long distances.

As a consequence, birch pollen has high impact on human health and the Earth’s climate. Hence, it is important to detect and characterize pollen as part of PBAPs. Over the last decades, extensive research activities focused on the detection and characterization of PBAPs using various techniques including off-line measurements such as chromatography and microscopy as well as cultivation of viable microorganisms [2]. Most biological particles show auto-fluorescent properties and therefore the focus of current studies is placed on fluorescence-based in situ measurements to investigate PBAPs concentrations with temporal resolution [25–28]. Fluorescence of biological cells is caused by fluorophores such as certain amino acids (tryptophan, *λ*_ex_/*λ*_em_ 280/348 nm; tyrosine, *λ*_ex_/*λ*_em_ 275/303 nm; phenylalanine, *λ*_ex_/*λ*_em_ 255/282 nm), nucleic acids, some co-enzymes (NADH, *λ*_ex_/*λ*_em_ 340/450 nm; FAD, *λ*_ex_/*λ*_em_ 450/520 nm; etc.), and others [29]. To detect PBAPs, laser-induced fluorescence using e.g. the wide band integrated bioaerosol spectrometer (WIBS) is widely applied by atmospheric and medical researchers. Gabey et al. [30] for instance measured high fluorescent particle concentrations over the canopy of a tropical forest. Fluorescence signals showed high intensities in the regions of tryptophan (*λ*_ex_, 280 nm) and NADH (*λ*_ex_, 370 nm), which are now commonly used in WIBS analysis [31]. However, biological particles consist of a variety of substances (proteins, carbohydrates, flavanols, etc.) potentially affecting the fluorescence quantum yield from fluorophores and possibly leading to quenching effects. Pöhlker et al. [32] reported fluorescence spectroscopy data of different pollen, including birch (*Betula pendula*), as a fingerprint for those species, in solid state and in solution. Furthermore, Hernandez et al. [31] investigated aerosolized pollen using a single-particle fluorescence sensor. In both studies, birch pollen spectra reveal that the typical protein signals (*λ*_ex_ 280 nm) are missing or are rather low despite the reported proteinaceous allergen presence.

Therefore, in this study, we tested the hypothesis that the fluorescence intensity of proteins from birch pollen is distorted within its native matrix. We employed UV/Vis spectroscopy, capillary zone electrophoresis (CZE), liquid chromatography (LC), electrospray ionization mass spectrometry (ESI–MS), and tandem MS (MS^2^ and MS^3^) to target major components in birch pollen washing water (BPWW, aqueous extract of birch pollen) possibly quenching the fluorescence activity of proteins and thus explaining the lack of corresponding protein fluorescent signals. Ultimately, defining the composition of BPWW will help to better characterize PBAPs in the future.

## Materials and methods

### Chemicals

Boric acid (≥ 99.5%), benzoic acid (≥ 99.0%), and dimethyl sulfoxide (99.9%) were purchased from Sigma Aldrich (St. Louis, MO, USA). Ultra-pure water was of Millipore® grade (specific resistivity 18.2 MΩcm (25 °C)). Sodium hydroxide pellets (99.5–100.5%), LiChrosolv water for chromatography (≥ 99.0%), and formic acid (98.0%) were obtained from Merck (Darmstadt, Germany), acetonitrile (99.9%) from VWR (Radnor, PA, USA), and sodium dodecyl sulfate (≥ 99.5%) from AppliChem (Darmstadt, Germany). Birch pollen grains (*Betula pendula*) were purchased from Thermo Fisher Scientific (Waltham, MA, USA), quercetin-3-O-sophoroside from Carbosynth (Newbury, UK), and Betv1a from Biomay (Vienna, Austria).

### Sample preparation

An aqueous extract of birch pollen powder was prepared (BPWW); 500 mg pollen was carefully blended with 10 mL ultra-pure water at room temperature. The mixture was left for 6 h and shaken occasionally two to three times. The suspension was then centrifuged for 5 min at 1.120 *rcf* at room temperature. The supernatant was decanted and filtered with a 0.2 μm cellulose acetate syringe filter (VWR, Radnor, PA, USA). The obtained BPWW was used for UV/Vis and fluorescence spectroscopy measurements.

For the fluorescence study, we enriched proteins present in birch pollen (birch pollen proteins, BPP) by filtering BPWW with a 10 kDa Nanosep® centrifuge cut-off filter (Pall, Port Washington, NY, USA). Therefore, 400 µL BPWW was pipetted into the spin tube filter and centrifuged. The retentate (50 µL) was washed with 400 µL ultra-pure water three times respectively. 50 µL retentate was recovered from the membrane and ultra-pure water was added to reach a total volume of 400 µL.

Prior to CZE and LC measurements, BPWW was treated with solid phase extraction (SPE) to avoid clogging the capillary or the LC column with C_18_ affine material. First, the stationary SPE phase (C_18_ Supelco Discovery®, Sigma Aldrich, St. Louis, MO, USA) was conditioned with 1.5 mL ultra-pure water. The liquid passed through the cartridge by applying vacuum. It was ensured that the sorbens did not run dry. To obtain the flow through, BPWW was diluted with ultra-pure water (1:3, resulting in *c* = 16.67 mg/mL) and loaded onto the cartridge. It was allowed to pass the bed with a speed of approx. one drop per second. The flow through was collected in a plastic vessel and the solvent was completely evaporated using a vacuum centrifuge for 5 h. The remaining residue was dissolved in 250 μL ultra-pure water. The obtained solutions were then used for CZE and LC analysis.

### Analysis

#### Fluorescence spectroscopy

Fluorescence spectra were recorded on a FPS920 laboratory-based fluorimeter (Edinburgh Instruments, Kirkton Campus, UK) including a Xe-900 xenon arc lamp (450 W) and a S900 single photon photomultiplier detection system. Excitation-emission maps (EEMs) were measured in a range of 240–400 nm/320–500 nm (*λ*_ex_/*λ*_em_) in 2 nm steps. To avoid first- and second-order excitation, we used an offset of 10 nm. In addition, emission scans were recorded between 320 and 400 nm at *λ*_ex_ = 280 nm using a 295 nm low-pass filter (Stablife Technology®, Newport, USA). Samples were measured in a quartz glass cuvette (500 µL, Hellma Quarz (Suprasil®), Müllheim, Germany). Prior to measurements, an excitation correction scan was performed to minimize the influence of the xenon lamp on the measurements of our samples.

#### Capillary zone electrophoresis

CZE measurements were performed on an Agilent 3DCE instrument (Agilent Technologies, Waldbronn, Germany). For the analyses, a fused silica capillary (Agilent Technologies) with an internal diameter of 50 μm and an outer diameter of 360 μm was used (*L*_tot_/_eff_ = 60.1/51.7 cm). During measurements, the temperature was kept at 22.5 °C and the electric field strength was 33 kV/m. 200 mM boric acid with 10 mM SDS (pH 8.4, adjusted with NaOH) was used as a background electrolyte (BGE). Analytes were detected at 205 nm with an UV/Vis detector. Prior to measurements, the capillary was conditioned with BGE for 5 min, post-conditioning included flushing with 1 M NaOH and ultra-pure water, 5 min each. As internal standards, benzoic acid (400 μg/mL stock in ultra-pure water diluted 20-fold in samples) and DMSO (5.5 mg/mL stock in ultra-pure water — electroosmotic flow (EOF) marker — diluted 20-fold in samples) were used. Samples (tenfold dilution of the obtained SPE flow through) were injected hydrodynamically (40 mbar, 8 s).

#### Liquid chromatography-electrospray ionization-mass spectrometry (LC-ESI-MS, ESI-MS^n^ (n = 2,3))

LC–ESI–MS analyses were performed on a Hitachi LaChrom Elite® LC-System equipped with an UV detector, a L2200 autosampler module, and a L2300 column oven (Merck Hitachi, Tokyo, Japan) coupled with an orthogonal ESI source of an Esquire 3000^plus^ ion trap mass spectrometer (Bruker Daltonics, Bremen; Germany). A C_18_ column (LiChrospher 100 RP18 endcapped, 125 × 2 mm, 5 µm, 100 Å, Merck, Darmstadt, Germany) was used. Mobile phase A was LiChrosolv water for chromatography and mobile phase B acetonitrile (ACN), both containing 12 mM formic acid (FA). The injection volume was set at 10 μL, flow rate 0.4 μL/min, and the temperature was 30 °C. The following gradient program was used for analyses: *t* = 0 min, 95% A, *t* = 2 min, 95% A, *t* = 17 min, 5% A, *t* = 22 min, 5% A, *t* = 24 min, 95% A, *t* = 30 min, 95% A. Mass spectra were acquired in positive and negative ion mode in two consecutive LC separations. All MS data were recorded in scan mode. The following settings for the ESI source and ion trap were used: (a) positive ion mode: nebulizer gas pressure: 1.17·10^5^ Pa, dry gas flow: 12.0 L/min, ion source dry temperature: 300 °C, scan range: *m/z* 100–2.000, spray voltage: 4.5 kV, collision gas: helium, maximum accumulation time: 400 ms, and (b) negative ion mode: nebulizer gas pressure: 0.69·10^5^ Pa, dry gas flow: 10.0 L/min, ion source dry temperature: 300 °C, scan range: *m/z* 50–1.500, spray voltage: 4.5 kV, collision gas: helium, maximum accumulation time: 200 ms. In addition, infusion ESI-MS^2^ and ESI-MS^3^ experiments, where selected masses are isolated and then excited for collision-induced dissociation (CID), were performed in positive and negative ion mode using helium as a collision gas. The fragmentation amplitude was between 0.2 and 1 V.

#### UV/Vis spectroscopy

UV/Vis spectra were recorded on an Implen Nanophotometer® Classic (Implen, Munich, Germany) which incorporates a xenon flash lamp, a dual channel Czerny Turner monochromator, and a CCD detector. For the fluorescence study, a UV-1600 PC (VWR, Radnor, USA) was used which is equipped with a deuterium and tungsten lamp.

## Results and discussion

### Fluorescence measurements

**Fig. 1:**
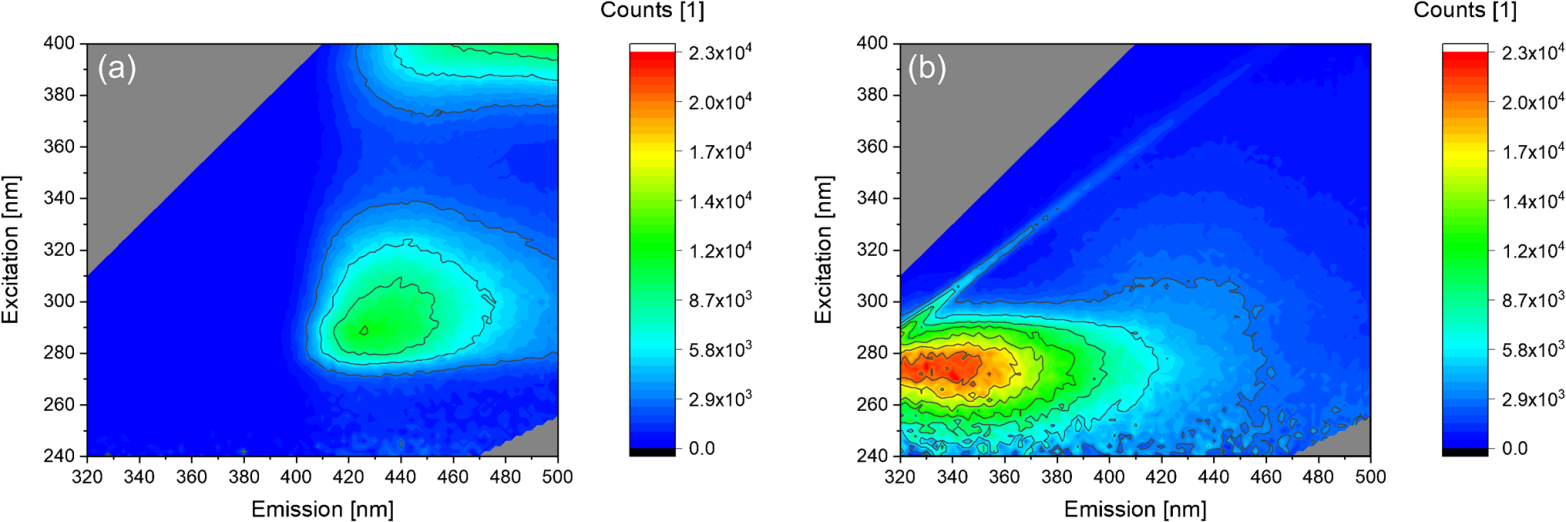
Fluorescence excitation-emission maps of **a** BPWW and **b** BPWW 1:100 [v:v] diluted with ultra-pure water. The diagonal faint line in **b** corresponds to the raman scattering signal of water [33]

Focusing on a standard method for PBAP characterization, we investigated the fluorescence behavior of BPWW. As the proteinaceous allergy elicitor Betv1a is water soluble, we expected corresponding fluorescence emissions when excited at 280 nm. However, when applying fluorescence spectroscopy to concentrated BPWW in a wavelength range including the typical protein region, unexpectedly, we did not observe any protein signals but instead obtained a maximum at *λ*_ex_ 270–310/*λ*_em_ 410–450 nm (Fig. 1a). In contrast, typical maxima for proteins would rather be recorded at emission wavelenghts between 280 and 340 nm.

Surprisingly, when diluting BPWW (1:100 [v:v] with ultra-pure water), the recorded maximum shifts to the expected value of *λ*_ex_ 250–290/*λ*_em_ 320–400 nm (Fig. 1b). Consequently, we reasoned that BPWW contains one or more substance(s) affecting the fluorescence quantum yield and inducing quenching and/or inner filter effects. Upon sample dilution, these effects are significanlty reduced. Moreover, it is possible that the concentration of proteins in BPWW leads to aggregation conformation changes and thus to a decrease of fluorescence intensity as well.

In general, distortion of fluorescence occurs either due to inner filter effects or quenching, which occurs either statically or dynamically [34]. The static mechanism describes the formation of a non-fluorescent complex between the fluorphore and the quencher. In contrast, dynamic quenching occurs due to the collision of the fluorophore with the quencher. In both cases, the excited fluorophore returns to the ground state emitting no or a rather low amount of photons [34]. Therefore, in a next step to identify the substance(s) inducing the quenching or filter effects of fluorescence emission in birch pollen, we analyzed BPWW with CZE and LC–MS.

### Separation of BPWW components

**Fig. 2:**
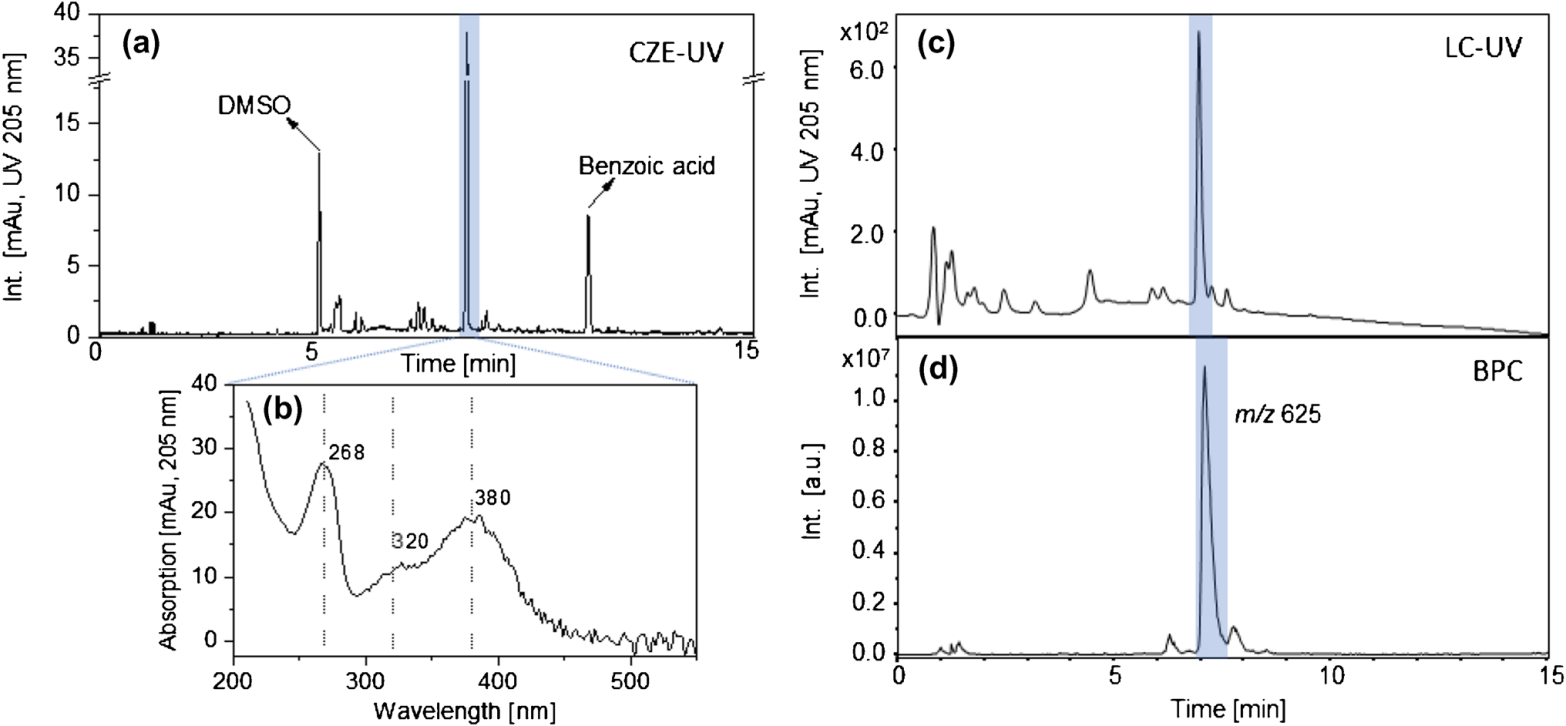
**a** Electropherogram of the SPE flow through of BPWW in an uncoated fused silica capillary at pH 8.4, BGE: 200 mM sodium borate with 10 mM SDS, id/od: 50/360 μm, *L*_tot_: 60.1 cm, *L*_eff_: 51.7 cm, field strength: 33 kV/m, temperature: 22.5 °C, UV detection at 205 nm, injection: 40 mbar pressure for 8 s. **b** UV/Vis spectrum of the substance migrating at 8.4 min. **c** UV chromatogram of BPWW flow through. A C_18_ column (LiChrospher 100 RP18 endcapped, 125 × 2 mm, 5 µm, 100 Å, Merck, Darmstadt, Germany) was used. Gradient elution using water and ACN, each containing 12 mM FA, at a flow rate of 0.4 μL/min. **d** Base peak chromatogram (BPC) of the BPWW flow through (negative mode)

The electropherogram of BPWW reveals one highly UV absorbing substance at a migration time of 8.4 min corresponding to an electrophoretic mobility *µ* = 20.9 ± 0.2 · 10^−9^ m^2^/Vs (Fig. 2a). The respective UV/Vis spectrum shows maxima at 268, 320, and 380 nm (Fig. 2b). Comparable to CZE measurements, the UV chromatogram (Fig. 2c) shows one strongly absorbing compound (measured at 205 nm UV absorption) at a retention time of 7.3 min. For both methods, the highlighted peak has an intensity around 10 times higher than the remaining peaks. A similar pattern was also found for the base peak chromatogram — a prominent peak with *m/z* 625 was recorded, however, slightly shifted to a later retention time, compared to the UV chromatogram. This marginal difference is caused by the connection tube between the LC and mass spectrometer and a resulting longer migration distance. Nevertheless, due to comparable UV/Vis absorption patterns as well as comparable migration times, we conclude that the highlighted signals represent the same substance.

### Mass spectrometric elucidation of highly UV active BPWW component

Since highly absorbing substances have the potential to interfere with fluorescence measurements, we focused on elucidating the strong UV/Vis absorbing component of BPWW. Seutter von Loetzen et al. [11] reported that the pigment quercetin-3-O-sophoroside (Q3OS) (MW 626 g/mol) is a natural ligand to the allergen Betv1a (more precisely to the major isoform Bet v 1.0101) in birch pollen. The full physiological function is still not clarified; however, Q3OS is thought to protect the pollen’s DNA from solar UV irradiation [10, 35]. In literature, quercetin and its derivatives revealed to provide plant tissues tolerance against several biotic and abiotic stresses including UV radiation [36].

**Fig. 3:**
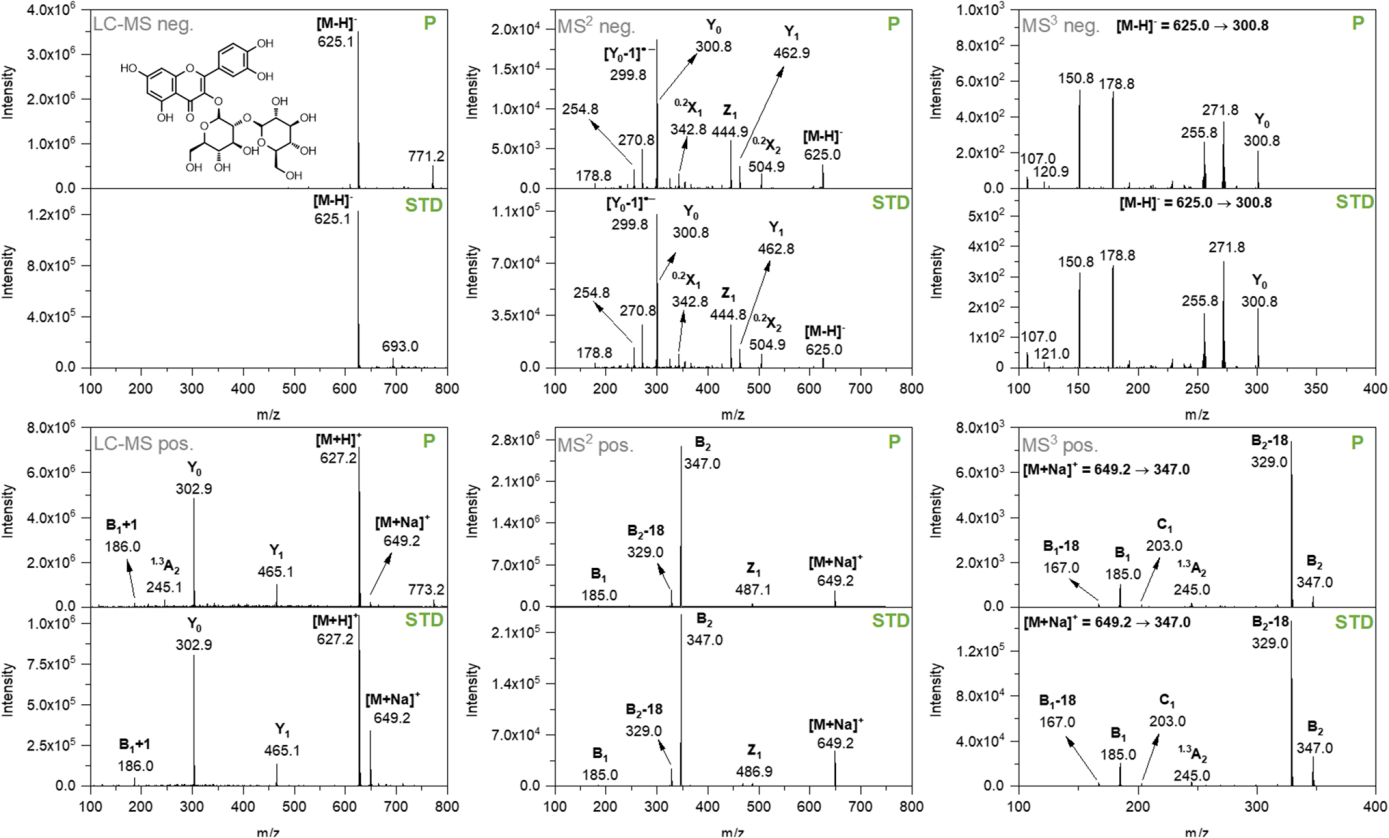
Comparison of LC–ESI–MS-, MS^2^-, and MS^3^-spectra of birch pollen washing water (P) and quercetin-3-O-sophoroside standard (STD) conducted in both positive and negative ion mode. The carbohydrate product ion nomenclature used is in accordance with Domon and Costello [37]

LC–MS data of this study nurtured the assumption that the strongly absorbing component of BPWW is the flavonoid Q3OS (negative ion mode *m/z* 625.1, positive ion mode *m/z* 627.2). To test this hypothesis, we conducted a control experiment. Therefore, Q3OS was purchased, extracted with ultra-pure water, and analyzed in analogy to BPWW using CZE, LC–MS, ESI-MS^2^, and ESI-MS^3^ in both negative and positive ion mode. Results for the latter methods are shown in Fig. 3.

**Fig. 4:**
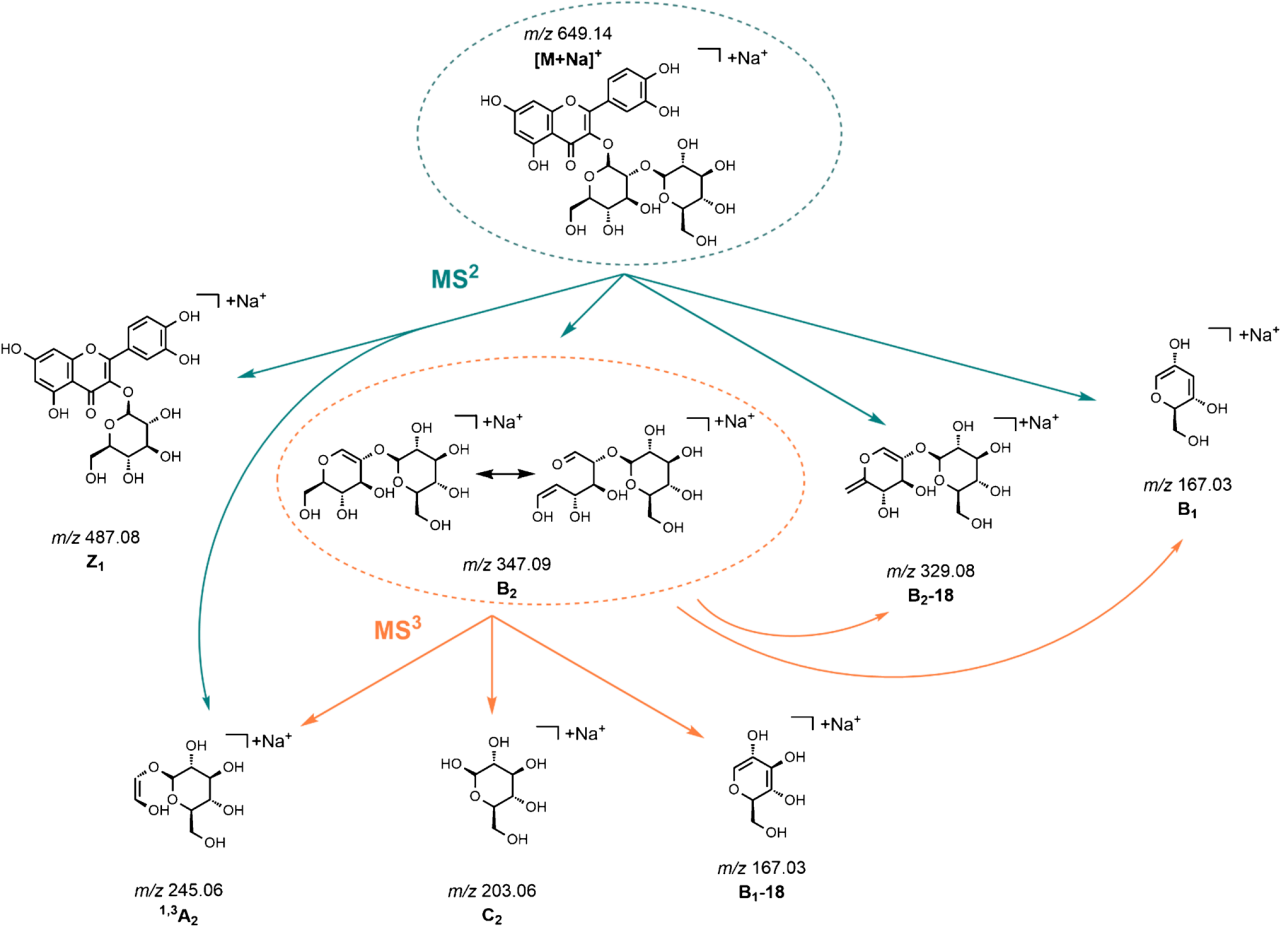
General fragmentation tree of [M + Na]^+^ precursor ion of quercetin-3-O-sophoroside in *B. pendula* birch pollen. The carbohydrate product ion nomenclature used is in accordance with Domon and Costello [37]

In the positive ion mode LC–ESI–MS experiment, the protonated molecule [M + H]^+^ *m/z* 627.2, the sodium adduct [M + Na]^+^ *m/z* 649.2, and fragment ions (Y_1_, Y_0_, B_1_ + 1, ^1,3^A_2_) were observed for BPWW (P) as well as for Q3OS (marked as STD, standard). The fragment ion Y_0_ was obtained as a result of the loss of two sugar moieties and indicates the aglycone in the positive mode. The fragment ion Y_1_ was formed from the loss of a hexose sugar moiety. In addition, the occurrence of the fragment ions B_1_ + 1 and ^1,3^A_2_ was also detected. For the MS^2^ experiments in positive ion mode, NaCl was added to the sample solution in order to improve ionization. The sodiated precursor ion [M + Na]^+^ *m/z* 649.2 and corresponding product ions (Z_1_, B_2_, B_2_-18, B_1_) were detected. The precursor ion Z_1_ *m/z* 487.1 was formed by a loss of a hexose. The detected B_2_ *m/z* 347.0 precursor ion is formed from the loss of the aglycone. Additionally, B_2_-18 (loss of a water molecule) and B_1_ precursor ions were observed. In the MS^3^ positive ion mode analysis of B_2_, the following product ions were detected: B_2_-18, ^1,3^A_2_, C_2_, B_1_, and B_1_-18. The fragments of all tandem-MS experiments in positive ion mode are summarized in Fig. 4.

**Fig. 5:**
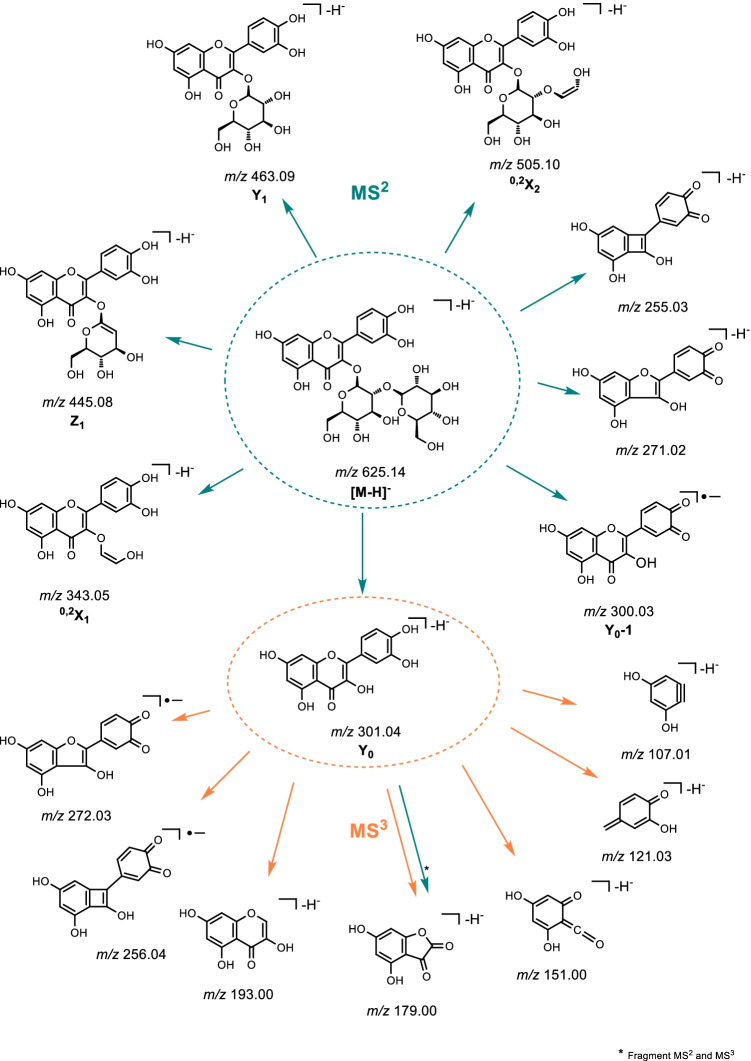
General fragmentation tree of [M-H]^−^ precursor ion of quercetin-3-O-sophoroside in *B. pendula* birch pollen. The carbohydrate product ion nomenclature used is in accordance with Domon and Costello [37]

In the negative ion mode LC–ESI–MS experiment for BPWW as well as Q3OS, the deprotonated molecule [M-H]^−^ *m/z* 625.1 was detected. The MS^2^ negative ion mode analysis shows the precursor ion [M-H]^−^ *m/z* 625.0 and corresponding product ions (^0,2^X_2_, Y_1_, Z_1_, ^0,2^X_1_, Y_0_, Y_0_-1). In line with MS^2^ positive ion mode experiments, Y_1_ was formed from the loss of a hexose sugar moiety and Y_0_ from the loss of two, indicating the aglycone in the negative ion mode. The precursor ion Y_0_-1 is formed from homolytic cleavage of the glycosidic bond [38]. Additionally, ^0,2^X_2_, Z_1_, and ^0,2^X_1_ were detected. Product ions of MS^3^ negative ion mode analysis of the precursor ion Y_0_ m/z 300.8 (aglycone) are summarized in Fig. 5 (orange). The fragments obtained from all tandem-MS experiments in negative ion mode are shown in Fig. 5.

**Fig. 6:**
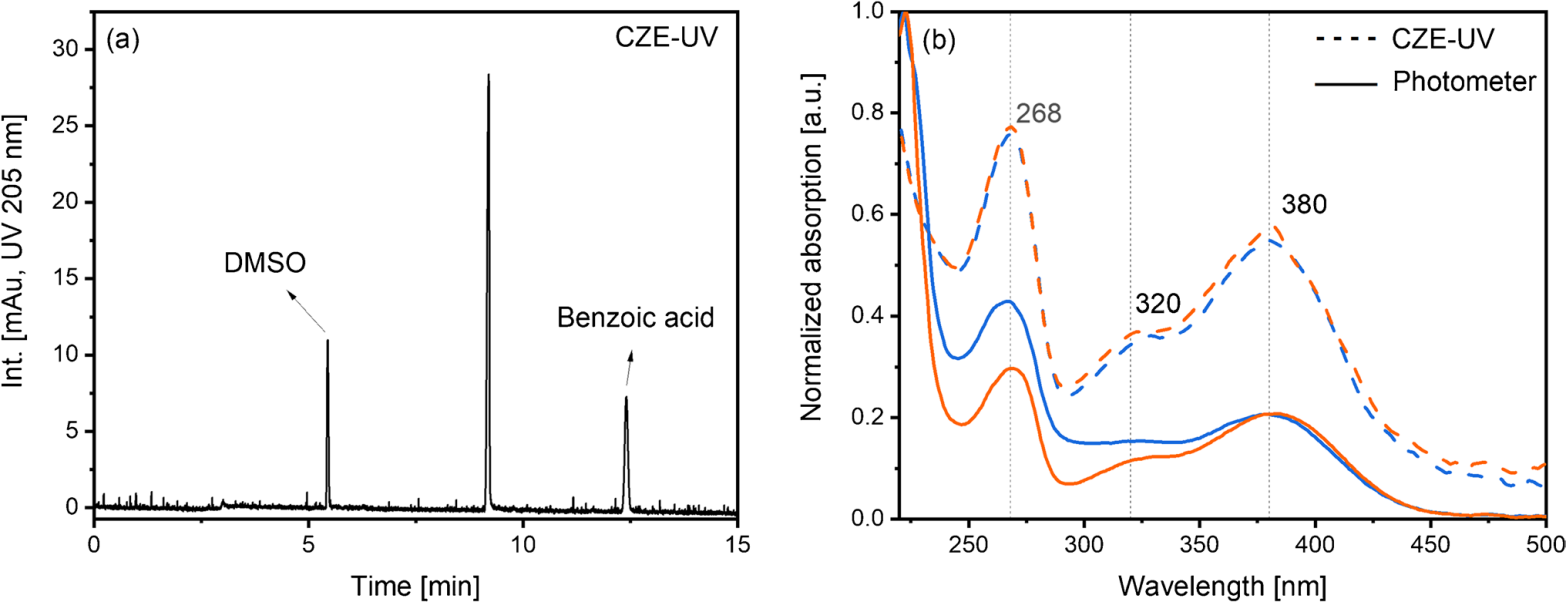
**a** Electropherogram of Q3OS. CZE conditions as in Fig. 2. **b** UV/Vis spectra of BPWW (orange) and Q3OS (blue). Dashed line: absorption spectrum recorded with CZE. Solid line: UV/Vis absorption spectrum recorded with the photometer

Besides matching MS data of BPWW with Q3OS, CZE migration data as well as absorption spectra recorded with the CZE and the UV/Vis photometer instrumentation were compared (Fig. 6). For CZE, electrophoretic mobility values of the main BPWW component (*µ* = 20.9 ± 0.2 · 10^−9^ m^2^/Vs, *n* = 3 measurements) matched well with the electrophoretic mobility of Q3OS (*µ* = 20.0 ± 0.1 · 10^−9^ m^2^/Vs, *n* = 3 measurements). Likewise, absorption spectra of BPWW and Q3OS (photometer), as well as the UV/Vis absorption spectra of the main BPWW component and Q3OS recorded with CZE, show absorption maxima at 268, 320, and 380 nm. Consequently, we elucidated Q3OS to be the main absorbing compound found in BPWW.

### Q3OS suppresses Betv1a/BPP fluorescence

**Fig. 7:**
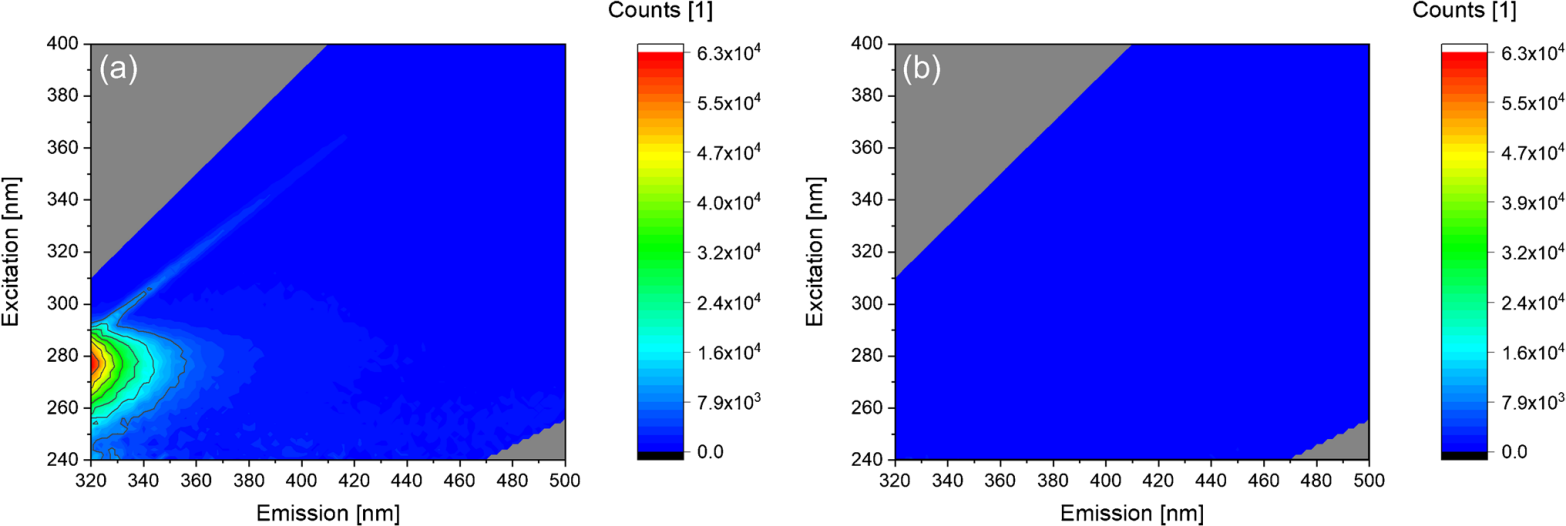
Fluorescence excitation-emission maps of **a** 5.7 µM Betv1a, and **b** 5.7 µM Betv1a and 320 μM Q3OS

Following these identification steps, we tested the hypothesis that Q3OS interferes with the fluorescence signal of birch pollen proteins and subsequently attempted to elucidate the corresponding effect. As a starting point, we recorded fluorescence EEM of (i) a standard protein, Betv1a (Fig. 7a) and (ii) purified BPP (Fig. S1a) which is the retentate of BPWW after centrifugal filtration (cut-off 10 kDa — Q3OS went into the filtrate). As expected, the assigned protein maximum at *λ*_ex_ 250–285/*λ*_em_ 320–350 nm is clearly visible in both samples. Further, as hypothesized, the protein maxima disappear, when Q3OS was added to the Betv1a/BPP solution (Fig. 7b, Fig. S1b), indicating the distortion of fluorescence signals.

**Fig. 8:**
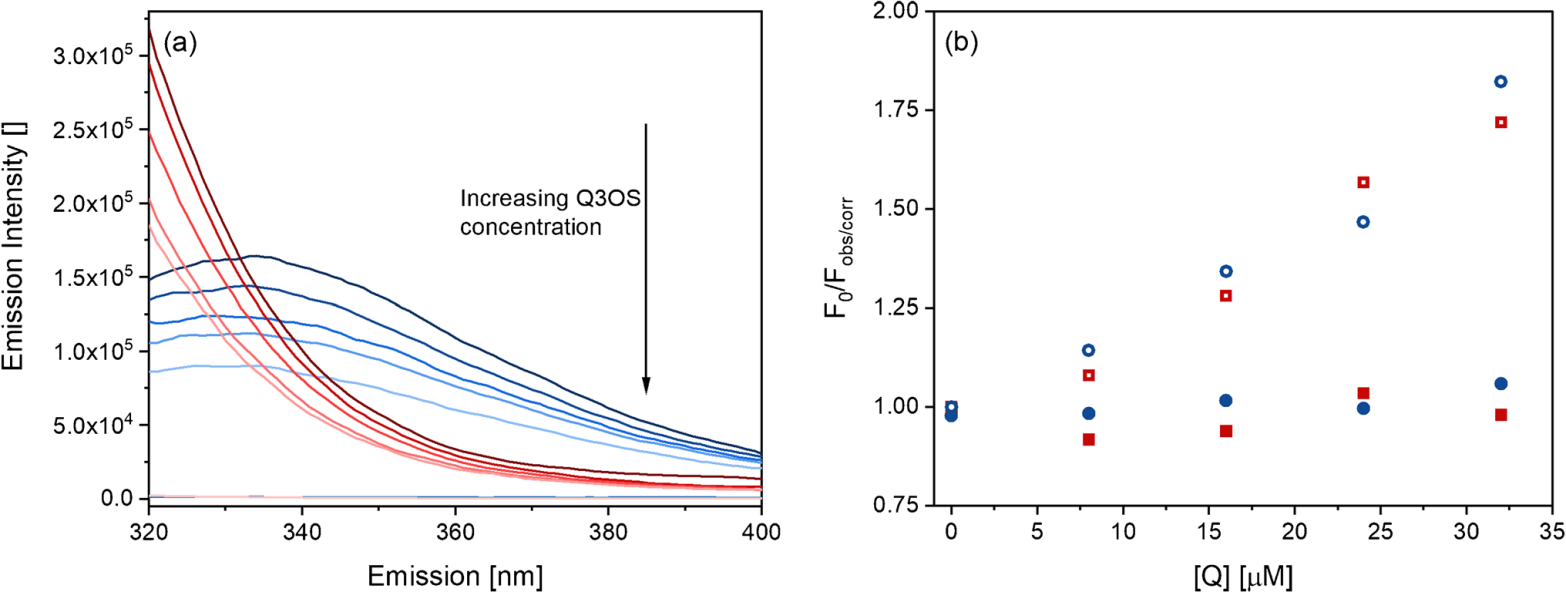
**a** Emission spectra of 1.1 μM Betv1a (red) and BPP (blue) in the presence of Q3OS with varying concentrations (0, 8, 16, 24, 32, and 320 μM) from dark to bright colors. Fluorescence spectra were recorded at *λ*_ex_ = 280 nm, pH 7.0, and T = 25 °C. **b** Stern–Volmer plot of Betv1a at *λ*_em,max_ = 320 nm (red circles) and BPP at *λ*_em,max_ = 334 nm (blue circles). Hollow symbols correspond to the raw data and filled symbols after the data was corrected for the inner filter effect

Further, we recorded fluorescence emission scans between 320 and 400 nm (*λ*_ex_ = 280 nm) of Betv1a and BPP in the presence of Q3OS with varying concentrations (Fig. 8a) as well as absorption spectra, respectively (see Appendix Fig. S2). The protein concentrations were kept constant. The emission scans of Betv1a (red) and BPP (blue) in Fig. 8a show strong fluorescence intensities. The spectrum on top (dark color) represents the corresponding proteins in absence of Q3OS. With increasing Q3OS concentrations (8, 16, 24, 32, 320 μM), the fluorescence intensities in the proteinaceous region of both Betv1a and BPP decrease. The addition of 320 μM Q3OS to the protein solutions leads to a total elimination of the fluorescence quantum yield. The maxima of BPP (*λ*_em_ = 334 nm) did not shift with the addition of Q3OS, indicating that the sample environment and thus the protein conformation did not change with increasing Q3OS concentrations [39, 40]. Due to limited set-up conditions, the emission maxima of Betv1a, which is below 320 nm, is not visible in the emission plots. In general, there are three phenomena that can explain the change in fluorescence in the presence of other compounds in the sample matrix: The occurrence of (i) an inner filter effect, which can be described as the co-absorption of the exciting light and/or re-absorption of the emitted light by a chromophore which is not the analyte [41], (ii) collisional quenching, where the energy of the excited fluorophore is transferred to a quencher molecule by colliding with it, facilitating non-radiative transitions to the ground state [34], and (iii) binding-related quenching where ground-complex formation, excited-state quenching in the complex, and binding induced structural changes are the reasons for the change in fluorescence intensity [42].

The Stern–Volmer equation describes the mechanism of collisional quenching:

1 $$\frac{{F}_{0}}{F}=1+{K}_{q}{\tau }_{0}\left[Q\right]=1+{K}_{SV}\left[Q\right]$$

where *F*_0_ is the fluorescence intensity in absence of the quencher, *F* the fluorescence intensity in the presence of the quencher, *K*_*q*_ the quenching rate constant, *τ*_0_ the life time of the fluorophore in absence of the quencher, [*Q*] the concentration of the quencher, and *K*_*SV*_ the Stern–Volmer quenching constant [43].

For both Betv1a and BPP, *F*_0_/*F* increases with increasing Q3OS concentration (see Fig. 8b). However, accounting for an inner filter effect and correcting the data in accordance to Lakowicz [34] (see Appendix, Equation S1), the obtained fluorescence intensities remain similar with increasing Q3OS concentrations excluding collisional quenching being responsible for the loss of the signal. The Lakowicz correction is an approximation and assumes that the emission radiation is observed at the center of the cuvette. However, the similar course in the Stern–Volmer graph of two different protein samples (Betv1a vs. a mixture of proteins, BPP) supports the fact that the inner filter effect is the dominant mechanism. Each individual protein has fluorescent residues on different positions, consequently their different exposure should lead to different courses. The inner filter effect is divided into a primary and a secondary effect. The primary effect occurs when the excitation beam is attenuated by an absorber. When the emitted radiation is reabsorbed by the absorber/quencher, it is the secondary mechanism [26, 44]. The absorption spectra of Q3OS in ultra-pure water show strong absorption intensities at around 270, 320, and 380 nm. This points to the fact that Q3OS filters not only the excitation beam, which would excite amino acid fluorophores in absence of Q3OS, but also the emission signal of the proteins.

Previous studies have shown that certain proteins form complexes with polyphenols such as flavonoids. Seutter von Loetzen et al. [11] showed, for example, that Q3OS in birch pollen binds to the hydrophobic pocket of Betv1a. It is assumed that the complex formation of Q3OS and Betv1a is rather important for the rigidification and compactness of the 3D structure of Betv1, plays a crucial role to avoid self-pollination as well as protecting the pollen’s DNA from solar UV irradiation [35]. In their study, they used NMR and MS data to verify the ligand binding properties. Another paper supported the hypothesis of Q3OS binding to Betv1a using surface acoustic wave measurements [45]. Hence, in addition to the inner filter effect, Q3OS may also quench proteins in birch pollen in a statical manner. However, to prove this hypothesis, the performance of time-resolved measurements and subsequently the determination of the bimolecular quenching rate constants from the variation of fluorescence lifetimes are needed to shed light on the detailed mechanism. Thus, to completely understand all quenching mechanisms, more Q3OS-based fluorescence quenching studies are necessary, especially as the elucidation of Q3OS as well as the quenching study was conducted in solution. However, fluorescence depends not only on temperature, pH, and the presence of various molecules possibly interacting with each other but also on the aggregate state of the samples. In aerosol measurements, many PBAPs, however, are not necessarily suspended in water but rather dry. Thus, the quenching effect of Q3OS on dry airborne birch pollen likewise still needs elucidation. This is particularly true for measurements with bioaerosol analysers (e.g., WIBS, Poleno, and Rapid-E +), which preferentially detect solid particles. However, results of this study support the comprehension on analyzing proteins with fluorescence-based techniques and point out the challenge to measure complex biological systems with fluorescence spectroscopy.

## Conclusion

In this study, we confirmed the presence of Q3OS in birch pollen washing water using CZE, UV/Vis spectroscopy, LC–MS, and tandem MS (MS^2^, MS^3^). Furthermore, we were able to show that Q3OS distorts the fluorescence signals of proteins present in birch pollen predominantly by inner filter effects as investigated on pure Betv1a as well as purified birch pollen proteins. However, based on the observation of previous studies, the occurrence of static quenching cannot be excluded as Q3OS forms a complex with Betv1a [11, 45]. The observation of changes in fluorescence signals of birch pollen, as shown by us, reveals the importance to critically scrutinize obtained fluorescence spectra when analyzing and detecting PBAPs. Precise and corrected fluorescence spectra are a prerequisite for (sub)pollen analytics in the atmosphere.

## Supplementary information

Below is the link to the electronic supplementary material.

Supplementary file1 (DOCX 43 KB)
