# Supplementary material for: Fluorescence signal of proteins in birch pollen distorted within its native matrix: Identification of the fluorescence suppressor quercetin-3-O-sophoroside
Source: Anal Bioanal Chem. 2022 May 12;414(25):7531–42. doi: 10.1007/s00216-022-04109-0 (PMC9482913; doi:10.1007/s00216-022-04109-0)
Supplement: Supplementary file 1 — Supplementary file1 (DOCX 43 KB) [file 216_2022_4109_MOESM1_ESM.docx]

**Appendix**

**
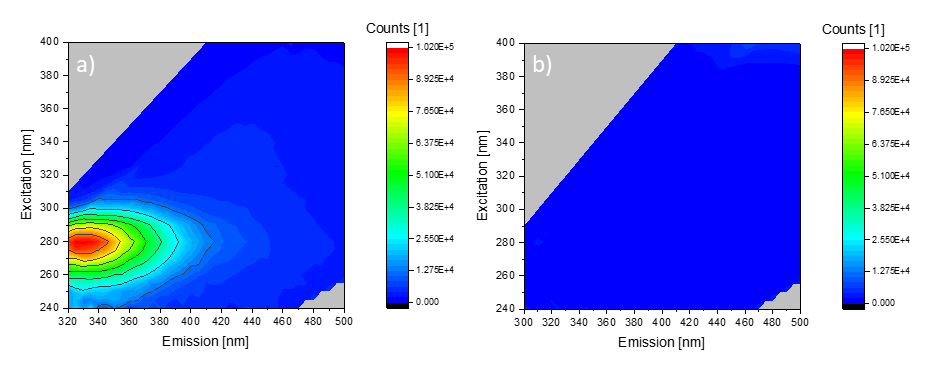
**

**Fig.S1:** Fluorescence excitation-emission maps of **a)** pure BPP and **b)** BPP in the presence of 320 μM Q3OS.


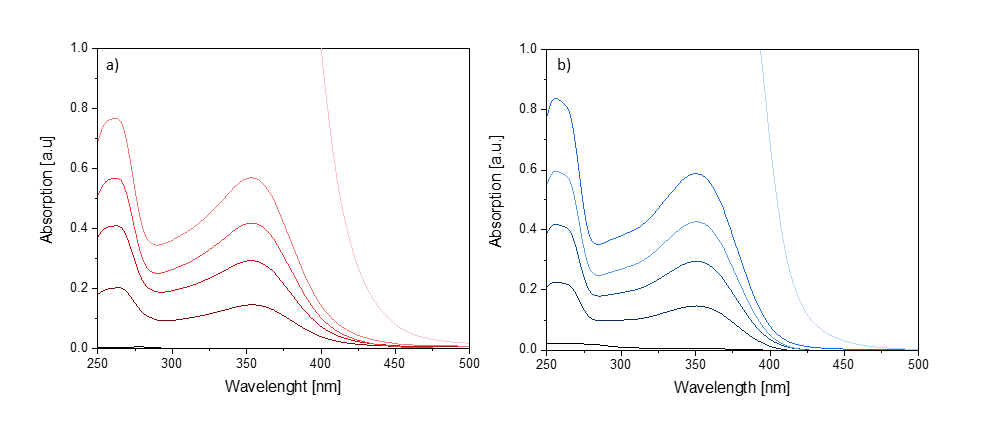


**Fig.S2:** Absorption spectra of **a)** 1.1 μM Betv1a and **b)** BPP in the presence of Q3OS with varying concentrations (0, 8, 16, 24, 32 and 320 μM) from dark to bright colours.

**Inner filter effect correction:**

| $F_{obs}=F_{corr}\cdot{10}^{-\frac{A_{ex}d_{ex}}{2}-\frac{A_{em}d_{em}}{2}}$ | (S1) |
| --- | --- |

*F_obs_* is the observed fluorescence, *F_corr_* the corrected fluorescence intensity that would be measured in the absence of inner-filter effects, *d_ex_* and *d_em_* the cuvette pathlength in the excitation and emission direction (in cm), respectively, and *A_ex_* the absorption intensity at the excitation wavelength of the absorber, *A_em_* the absorption intensity at the emission wavelength [1, 2].

**References**

1. Lakowicz JR. Principles of Fluorescence Spectroscopy. 3rd ed.: Springer US; 2006. p. 689.

2. Van de Weert M, Stella L. Fluorescence quenching and ligand binding: A critical discussion of a popular methodology. Journal of Molecular Structure. 2011;998(1-3):144-50. doi:<https://doi.org/10.1016/j.molstruc.2011.05.023>.
